# Influence of Redox Imbalances on the Transposition of Insertion Sequences in *Deinococcus geothermalis*

**DOI:** 10.3390/antiox10101623

**Published:** 2021-10-15

**Authors:** Qianying Ye, Chanjae Lee, Eunjung Shin, Sung-Jae Lee

**Affiliations:** Department of Biology, Kyung Hee University, Seoul 02447, Korea; leavesying@khu.ac.kr (Q.Y.); or qkektk456@naver.com (C.L.); eunj@khu.ac.kr (E.S.)

**Keywords:** *Deinococcus geothermalis*, cystine importer, insertion sequences, oxidative stress, redox-balance, transcriptomic analysis, transposition

## Abstract

The transposition of insertion sequence elements was evaluated among different *Deinococcus geothermalis* lineages, including the wild-type, a cystine importer-disrupted mutant, a complemented strain, and a cystine importer-overexpressed strain. Cellular growth reached early exponential growth at OD_600_ 2.0 and late exponential growth at OD_600_ 4.0. Exposing the cells to hydrogen peroxide (80–100 mM) resulted in the transposition of insertion sequences (ISs) in genes associated with the carotenoid biosynthesis pathway. Particularly, IS*Dge7* (an IS*5* family member) and IS*Dge5* (an IS*701* family member) from the cystine importer-disrupted mutant were transposed into phytoene desaturase (*dgeo*_0524) via replicative transposition. Further, the cystine importer-overexpressed strain Δ*dgeo*_1985R showed transposition of both IS*Dge2* and IS*Dge5* elements. In contrast, IS transposition was not detected in the complementary strain. Interestingly, a cystine importer-overexpressing strain exhibited streptomycin resistance, indicating that point mutation occurred in the *rpsL* (*dgeo*_1873) gene encoding ribosomal protein S12. qRT-PCR analyses were then conducted to evaluate the expression of oxidative stress response genes, IS elements, and low-molecular-weight thiol compounds such as mycothiol and bacillithiol. Nevertheless, the mechanisms that trigger IS transposition in redox imbalance conditions remain unclear. Here, we report that the active transposition of different IS elements was affected by intracellular redox imbalances caused by cystine importer deficiencies or overexpression.

## 1. Introduction

Most biochemical pathways in cells involve redox reactions, which highlights the critical importance of redox balance in the maintenance of homeostasis. Redox reactions often involve molecules with thiol or sulfhydryl (-SH) functional groups, including low-molecular-weight (LMW) thiols, cystine-derived thiols, and redoxins [1,2]. Particularly, redox balance is largely regulated by the uptake of cysteine and cystine-derived thiol compounds from the environment through membrane channels, as well as the activity of specific importers. Additionally, this mechanism maintains the intracellular redox balance in Gram-positive and Gram-negative bacteria [3,4,5]. Reactive oxygen/nitrogen species (RONS) and reactive electrophilic species (RES) include quinones, aldehydes, and epoxides, which can damage many cellular components, including nucleic acids, lipids, proteins, and metal cofactors [2]. Particularly, reactive oxygen species (ROS), including H_2_O_2_, are known to alter protein structure, resulting in the activation of catalytic sites via the oxidation of cysteine residues, in addition to enzyme inactivation and DNA damage [6,7]. Therefore, many types of thiol compounds are known to counteract the adverse effects of oxidative stress caused by ROS such as hydrogen peroxide, hydroxyl radicals, and superoxide. Most cells produce LMW molecules to protect themselves against RONS and RES. For instance, eukaryotes and many Gram-positive bacteria synthesise glutathione (GSH) as their main thiol reductant. Moreover, some GSH-deficient microorganisms including archaea rely on LMW thiol reductants such as mycothiol (MSH) and bacillithiol (BSH) [2,8], suggesting that these thiol compounds are directly or indirectly involved in antioxidant responses, especially in redox-sensing regulator systems. Other gene regulation systems based on thiol redox-switches in microorganisms have also been reviewed by Antelmann and Helmann [6], Imlay [7,9], Hillion and Antelmann [10], Sevilla et al. [11], and Lee and Lee [12].

The members of the genus *Deinococcus* are known to tolerate extreme conditions such as radiation, desiccation, and oxidative stress, and are therefore widely used as models for the evaluation of survival strategies using redox control, DNA damage repair, and enzymatic and non-enzymatic responses [13,14,15,16,17,18]. Redox-based responses and the stress response mechanisms of the members of the genus *Deinococcus*, which contains over 60 species, have thus garnered much attention among the scientific community. Our study thus sought to evaluate the antioxidation mechanisms of *Deinococcus geothermalis*, a moderate thermophile that can survive at 48 °C [19,20]. In a previous study, a cystine importer was characterised in *D. geothermalis* DSM11300^T^, and the study largely focused on the changes in the amount of total intracellular thiol. The cystine importer was strongly induced (i.e., >60-fold up-regulation) at the stationary growth phase in the wild-type *D. geothermalis* strain [21]. A mutant strain lacking the cystine importer showed higher sensitivity to oxidative stress upon treatment with hydrogen peroxide; however, the complemented strain recovered its tolerance [21,22]. Therefore, the cystine importer likely plays a primitive role against oxidative stress that predates enzymatic defence. Further, the general oxidative stress response regulator OxyR was strongly induced under oxidative stress conditions but this reaction did not coincide with the expression of protecting enzymes such as catalase and superoxide dismutase (SOD) in *D. geothermalis* [21].

The prokaryotic genome contains several transposable elements such as insertion sequences (ISs), transposons (Tn), and short repeat elements, which have been abundantly detected by genomic surveys as components of the “mobilome” [23,24,25]. A typical bacterial IS element consists of one or two transposases (Tpase) and terminal inverted repeat (TIR) sequences. Additionally, direct repeat (DR) sequences are mainly produced during the genome integration of IS elements [26,27,28]. The *D. geothermalis* genome has 73 full-length ISs encompassing nine IS families including IS*1*, IS*4*, IS*5*, IS*6*, IS*66*, IS*200*/IS*605*, IS*630*, IS*701*, and IS*982*. Importantly, several of these IS families were actively transposed upon hydrogen peroxide treatment in two mutant strains with disrupted DNA-binding proteins [29,30].

A *dgeo*_0257 knock-out mutant was constructed in a previous study that sought to identify novel DNA protecting proteins (Dps) with the capacity to adsorb metals, especially ferrous ions. Under hydrogen peroxide-induced oxidative stress conditions, we selected a non-pigmented colony with a broken phytoene desaturase (*dgeo_*0524) caused by the integration of IS*Dge7* (a member of the IS*5* family) [29]. Additionally, hydrogen peroxide treatment resulted in the discovery of an IS transposition in a mutant strain where the LysR family transcriptional regulator *dgeo*_2840 gene was disrupted. This also interrupted the carotenoid biosynthesis pathway via the integration of IS*Dge6* (a member of the IS*5* family) [30]. Thus, Dps and LysR family deficient mutant strains exhibited specific IS element transposition in genes associated with the carotenoid biosynthesis pathway [31]. However, the transposition of specific IS type element was strictly limited by the frequency of mutant production.

Our study thus evaluated the effect of redox balance on the transposition of IS elements using a cystine importer deficient mutant (Δ*dgeo*_1986-87) and its complemented or overexpressed mutant strains (Δ*dgeo*_1986-87/pRADgro_1986-87 or Δ*dgeo*_1985R). Our findings demonstrated that the oxidation state of the cystine importer-disrupted mutant caused IS*Dge5* and IS*Dge7* to be actively transposed to other sites in the genome. In the reduction state, the IS*Dge2* and IS*Dge5* elements were transposed in the cystine importer-overexpressed mutant, resulting in a disruption of carotenoid biosynthesis genes. However, we have not identified any signs of IS transposition in the complemented strain of the cystine importer. Therefore, we hypothesised that the active transposition of ISs was modulated by the cystine importer through intracellular redox imbalance. Three elements (IS*Dge2*, IS*Dge5*, and IS*Dge7*) were found to be transposed in carotenoid pigment biosynthesis genes. Additionally, the cystine importer-overexpressing strain exhibited a streptomycin-resistant phenotype that was attributed to a point mutation in *rpsL*, which encodes the 30S ribosomal protein S12. This streptomycin resistance phenotype could also be used for the detection of IS transposition. Real-time quantitative reverse-transcription polymerase chain reaction (qRT-PCR) analysis of genes associated with the oxidative stress response, IS elements, and enzymes producing low-molecular-weights thiol compounds (MSH and BSH) was also performed. These experiments were conducted at two different growth phases, as well as in the presence and absence of hydrogen peroxide. Nevertheless, the mechanisms that trigger the induction of IS transposition in response to redox imbalance remain unclear.

## 2. Materials and Methods

### 2.1. Bacterial Strains and Culture Conditions

*D. geothermalis* DSM11300^T^ was obtained from the Korean Agricultural Culture Collection (KACC12208, http://genebank.rda.go.kr/ accessed on 15 April 2019). In previous studies, we also constructed a knock-out mutant of the cystine importer *dgeo*_1986-87 (Δ*dgeo*_1986-87) and a complemented strain containing a recombinant expression vector pRADgro::*dgeo_*1986-87 constructed via the ligation of a full-length DNA fragment containing the *dgeo*_1986-87 cystine importer. Additionally, the *dgeo*_1985R (Δ*dgeo*_1985R) mutant with a disrupted putative TrmB family regulator resulting in the overexpression of the cystine importer was also constructed via homologous recombination of the regions adjacent to the kanamycin-resistant gene [21,22]. The *Deinococcus* culture complex medium is a commonly used TGY medium consisting of 1% tryptone, 0.1% glucose, and 0.5% yeast extract. *D. geothermalis* wild-type and mutant strains were incubated overnight at 48 °C. The cystine importer-complemented strain was grown in TGY media containing 8 µg/mL of kanamycin or 3 µg/mL of chloramphenicol.

### 2.2. Non-Pigmented Colonies and Streptomycin-Resistant Selection

Wild-type and mutant strains were cultured in TGY medium. The cells were first allowed to reach an optical density (OD_600_) of 2.0 for the early exponential growth phase and an OD_600_ of 4.0 for the late exponential growth phase (approximately 2.95 ± 4.0 × 10^7^ cells) [21]. The cells were then centrifuged and resuspended in TGY medium and adjusted to an OD_600_ of 2.0. The samples were centrifuged again and resuspended with a 0.9% NaCl solution. Following treatment with hydrogen peroxide at a final concentration of 80 and 100 mM for 1.5 h, the samples were diluted and spread on TGY agar media with or without streptomycin and grown at 48 °C for two days. Non-pigmented colonies were isolated and serially diluted via streaking on a TGY agar plate for pure culture. Streptomycin-resistant mutant strains were directly selected from a TGY agar plate containing 50 µg/mL streptomycin for further pure cultivation.

### 2.3. Detection of Insertion Sequence Transposition

Genomic DNA was extracted from the cells using the HiYield™ Genomic DNA Mini Kit (Real Biotech Corporation, Taipei, Taiwan). Polymerase chain reaction (PCR) was performed using the ExTaq polymerase (TaKaRa, Maebashi, Japan) coupled with four primer sets specific to carotenoid biosynthesis genes, including *dgeo*_0523 for phytoene synthase, *dgeo*_0524 for phytoene desaturase, *dgeo*_0857 for lycopene cyclase, and *dgeo*_2309 for carotene synthase, as well as four streptomycin resistance genes to detect the locus where the IS transposition occurred [29]. Streptomycin resistance biomarkers were also evaluated using primers specific for rRNA-associated proteins and their modifying enzymes including *mthA* for a methylthioadenosine nucleosidase (*dgeo_*0447 and 0776), *rpsL* for a ribosomal protein S12 (*dgeo*_1873), and *rsmG* for a AdoMet-dependent methyltransferase (*dgeo*_2335). The PCR products were separated by electrophoresis in a 1% agarose gel, after which they were purified using the AccuPrep^®^ PCR Purification Kit (Bioneer, Daejeon, South Korea). DNA sequencing analysis of PCR products was performed by Macrogen Co. (Seoul, South Korea) via capillary electrophoresis sequencing using an ABI 3730xl system. The bacterial IS detection platform ISFinder (http://isfinder.biotoul.fr, (accessed on 15 April 2019)) was used to identify the transposed IS elements [32].

### 2.4. Transcriptomic Analysis by RNA-Seq

Transcriptomic analysis was conducted using RNA-Seq technology to quantify the expression levels of the entire transcriptome to determine the functional roles of the cystine importer in the Δ*dgeo*_1986-87 mutant examined in a previous study [21]. The transcript expression in the cystine importer-disrupted mutant was then compared to that of wild-type *D. geothermalis* at OD_600_ 4.0 (i.e., the late exponential growth phase). Total RNA was extracted using the RIBOEx reagent (GeneAll, Seoul, South Korea). The extracted total RNA was then purified using the RNeasy Mini Purification Kit (Qiagen, Hilden, Germany) coupled with the RNase-Free DNase I Set (Qiagen, Germany) [30]. After confirming the quality of the purified RNA, e-biogen Co. (Seoul, South Korea) sequenced the isolated total RNA with an Illumina HiSeq1000 sequencer. Data analysis was performed using the “ExDEGA” (Excel-based Differentially Expressed Gene Analysis) program at e-biogen Co. The expression level of each gene in the full genome was reported as log2 normalised read counts resulting from the comparison between ∆*dgeo*_1986-87 and the wild-type strain. RNA-Seq was performed using three mutant samples (Δ*dgeo*_1986-87, Δ*dgeo*_2840, and Δ*dgeo*_0257) and a wild-type strain sample. Moreover, constitutively expressed genes were tested via a two-sample *t*-test. Differences were deemed statistically significant when *p* < 0.05. The transcriptomic data of four samples including the cystine importer-disrupted ∆*dgeo*_1986-87 strain have been deposited in the NCBI Gene Expression Omnibus (GEO) database under accession number GSE151903 [31].

### 2.5. Quantitative Real-Time (qRT)-PCR

The RNA-Seq data of the target genes were validated by qRT-PCR. The cells were prepared at both OD_600_ 2.0 and 4.0. Samples were normalised to an OD_600_ of 2.0 with TGY medium, then harvested and resuspended with a 0.9% NaCl solution, followed by the addition of H_2_O_2_ to a final concentration of 50 mM. The samples were then incubated at 48 °C with shaking at 180 rpm for one hour and harvested by centrifugation at 4500 rpm and 4 °C for 15 min. Total RNA was extracted using the RNeasy mini purification kit (Qiagen, Germany) after phenol extraction and DNaseI treatment. The quality and quantity of the total RNA were then determined, and RNA amounts were normalised using a DS-11 spectrophotometer (Denovix Inc., Wilmington, DE, USA). After normalising the extracted RNA to 1 µg in an 8 µL volume, cDNA was synthesised using the PrimeScript^TM^ 1st strand cDNA Synthesis kit (TaKaRa, Maebashi, Japan) as described by Kim et al. [22]. qRT-PCR was then performed using TB Green^®^ Premix Ex Taq^TM^ (TaKaRa, Japan) on a CFX96^TM^ Optics Reaction Module (Bio-Rad, Berkeley, CA, USA) following standard laboratory protocols. The expression level of glyceraldehyde-3-phosphate dehydrogenase (GAPDH), which maintains a stable expression level in all growth phases, was used to normalise the relative expression level of each gene. Relative gene expression was calculated using the comparative threshold cycle (ΔΔCT) method [33]. The data were reported as the means and standard deviations (SDs) of three replicate experiments. Pairwise comparisons between sample groups were conducted via Student’s *t*-test using the Prism^TM^ software (ver. 8.0) and differences were considered statistically significant at *p* < 0.05–0.0001.

## 3. Results

### 3.1. Physiological Properties of Target Gene Disrupted and Complemented Mutants

**Figure 1:**
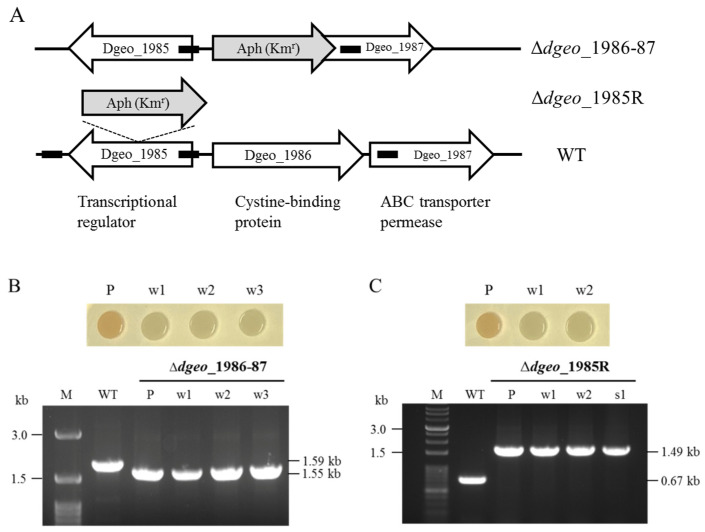
PCR confirmed both parent strains ∆*dgeo*_1986-87 and ∆*dgeo*_1985R, and their non-pigmented colonies induced by hydrogen peroxide, as well as the wild-type strain. Schematic illustration of mutant construction (**A**). Phenotypic difference between parent (p) and non-pigment strains (w) and PCR confirmation with primer set encompassing target genes for *dgeo*_1986-87 (**B**) and *dgeo*_1985R (**C**). Lanes: M, size marker; WT, wild-type; P, parent strain; w, non-pigment strains; s, streptomycin-resistant strain.

All experimental strains were confirmed via PCR detection (Figure 1). Growth curve analyses indicated that the cystine importer-disrupted mutant strain had lower maximum cell densities than those of the wild-type (Figure S1). However, the cystine importer-complemented and -overexpressed strains recovered from these low growth rates. The cystine importer-disrupted mutant strain exhibited higher susceptibility to oxidative stress than the wild-type *D. geothermalis* at the OD_600_ of 2.0 [E] growth stage in response to hydrogen peroxide treatment. The growth rate of the cystine importer-complemented and -overexpressed strains recovered at the [E] phase, and all tested strains exhibited similar susceptibility to H_2_O_2_ at the OD_600_ 4.0 [L] growth phase (Figure S2). The cystine importer-disrupted mutant recovered at the [L] phase from the growth inhibition caused by oxidative stress. Therefore, transcriptomic analyses were then conducted to identify dysregulated redox-associated genes.

### 3.2. Major Gene Categories Differentially Expressed in the Cystine Importer-Disrupted Mutant

Former laboratory members performed transcriptomic analyses using RNA-Seq to compare the gene expression levels between the wild-type strain and the cystine importer-disrupted mutant. Table 1 shows a list of the up-regulated genes with more than 3-fold induction (normalised RC log2 scale). Interestingly, some categories, including transposases of the IS*Dge5* element, GCN5 family acetyltransferase genes, MFS efflux transporters, ABC transporters, sigma factors, several transcriptional regulators, and various genes encoding enzymes, were specifically up-regulated in the ∆*dgeo_*1986-87 mutant strain. Down-regulated genes (<0.3-fold changes) were also identified, including 3-hydroxyisobutyrate dehydrogenase, thioesterase, pterin dehydratase, citrate synthase, peptidase, and ferric reductase, as well as a peptide ABC transporter gene (*dgeo*_2122), which was strongly down-regulated with a 0.07-fold expression (Table 2). Therefore, the cystine importer-disrupted mutant exhibited a unique impairment in substrate uptake and dysregulation of specific genes involved in several physiological function categories. Here, we propose that the active transposition of a particular type of IS element occurred due to changes in the intracellular redox balance.

### 3.3. Detection of IS Transposition

**Figure 2:**
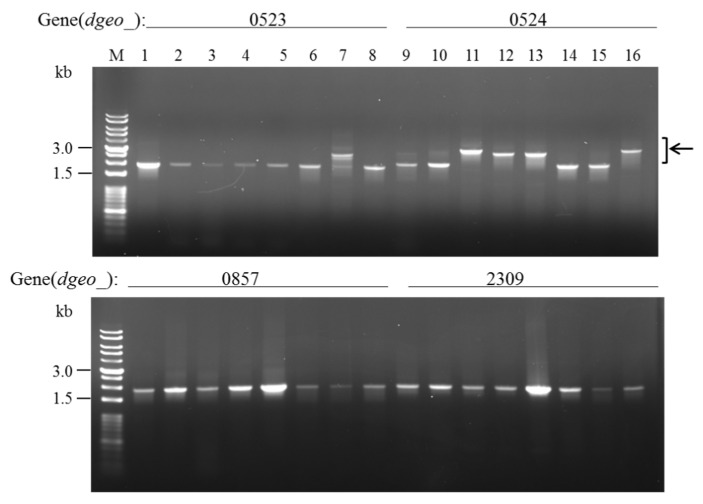
PCR detection of transposition loci on four target genes for carotenoid biosynthesis within *dgeo_0523* (1.87 kb), *dgeo*_*0524* (2.09 kb), *dgeo_0857* (1.85 kb), and *dgeo*_*2309* (1.86 kb). Lanes: M, size marker; 1 and 9, wild-type; 2 and 10, ∆*dgeo*_1986-87; 3 and 11, ∆*dgeo*_1986-87_w1; 4 and 12, ∆*dgeo*_1986-87_w2; 5 and 13, ∆*dgeo*_1986-87_w3; 6 and 14, ∆*dgeo*_1985R; 7 and 15, ∆*dgeo*_1985R_w1; 8 and 16, ∆*dgeo*_1985R_w2. Arrow indicates the IS integrated samples: 7, 2.62 kb; 11 and 16, 3.24 kb; 12 and 13, 2.96 kb.

Two and one non-pigmented mutant strains were respectively isolated from the Δ*dgeo*_1986-87 mutant strain (2.5 × 10^7^ cells) at OD_600_ 2.0 and 4.0 after hydrogen peroxide treatment at a final concentration of 80 mM for 1 h (Figure 1B). Further, all non-pigmented mutant strains from the Δ*dgeo*_1985R mutant (3.4 × 10^7^ cells) at OD_600_ 2.0 and 4.0 were isolated after hydrogen peroxide treatment at a final concentration of 100 mM for 1.5 h (Figure 1C). Therefore, the non-pigmented mutants were produced at a less than 2.98 ± 5.0 × 10^−8^ frequency. Based on a previous study that also evaluated IS transposition in non-pigmented mutant strains, four major carotenoid biosynthesis pathway-associated genes (*dgeo*_0523, *dgeo*_0524, *dgeo*_0857, and *dgeo*_2309) were amplified via PCR using a specific primer set for each gene [29,30]. IS integration into the target gene resulted in an enlarged PCR product reaching as much as the total length of the IS (Figure 2: lanes 7, 11–13, and 16 in *dgeo*_0523 and 0524, upper panel).

**Figure 3:**
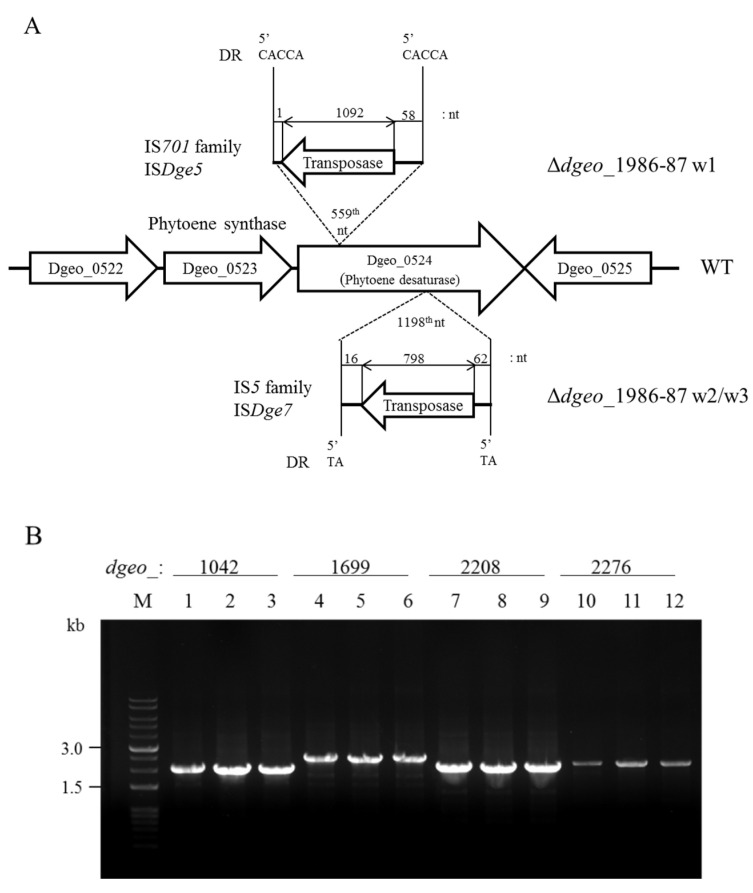
Detection of IS integration loci in three non-pigmented ∆*dgeo*_1986-87 mutant strains (w1-w3). (**A**) There were two IS element integration sites of IS*Dge5* and IS*Dge7* on *dgeo*_0524 encoding a phytoene desaturase. (**B**) PCR detection of four IS*Dge7* copies in the genome using the target gene primer sets from the wild-type, a parent strain, and ∆*dgeo*_1986-87_w2: *dgeo*_1042 (2.0 kb), *dgeo*_1699 (2.37 kb), *dgeo*_2208 (2.03 kb), and *dgeo*_2276 (2.11 kb). Lanes: M, size marker; 1, 4, 7, and 10, wild-type; 2, 5, 8, and 11, parent strain; 3, 6, 9, and 12, ∆*dgeo*_1986-87_w2 mutant. PCR detection of ten IS*Dge5* copies in the genome indicated in Figure S3.

Interestingly, all three Δ*dgeo*_1986-87 non-pigmented mutant strains exhibited *dgeo*_0524 encoding phytoene desaturase disruption through IS integration on two different sites. The integrated site of Δ*dego*_1986-87w2 and w3 was identical at the 119^8^^th^ nucleotide and the IS element IS*Dge7* of the IS*5* family member. IS*Dge7* resulted in a constant direct repeat (DR) sequence with ‘TA’, and its terminal inverted repeat sequence (TIR) was ‘GAGGCTGG’. Δ*dego*_1986-87w1 contained the IS*701* family member IS*Dge5* element, which was integrated at the 559^th^ nucleotide. The IS*Dge5* element resulted in a variable 5-nucleotides DR sequence ‘CACCA’ and the TIR sequence was ‘CTCAGGAGTTGCACCT’ (Figure 3A). To determine the transposition mechanisms, PCR-detection was performed with specific primer sets for all copies of IS members in the genome (Figure 3B, four copies for IS*Dge7* and Figure S3, ten copies for IS*Dge5*). All copies of IS members were amplified with identical sizes. Therefore, we concluded that the IS*Dge7* and IS*Dge5* elements were transposed via the copy-and-paste mode.

**Figure 4:**
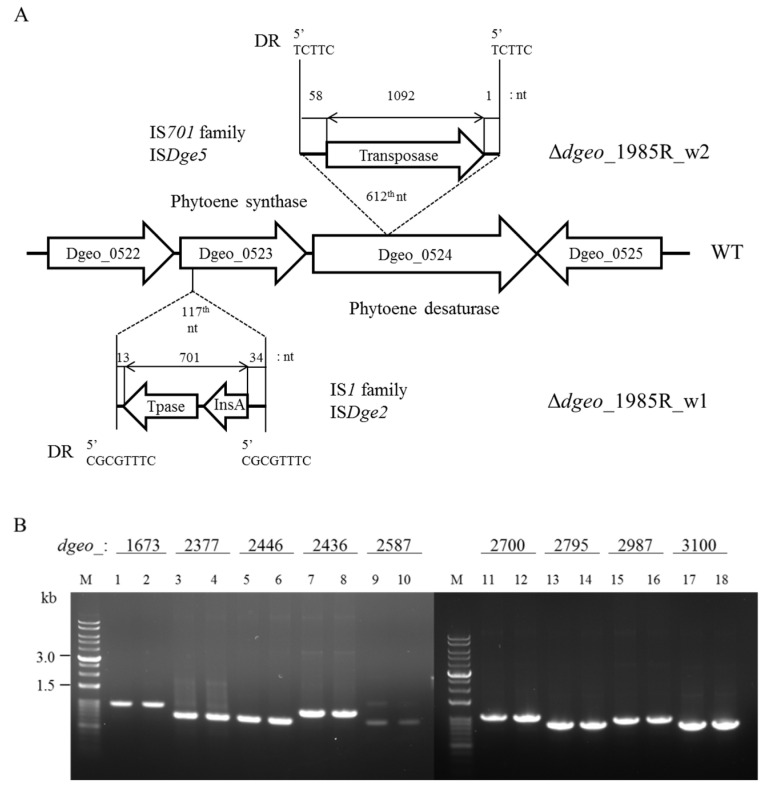
Detection of IS integration sites in two non-pigment ∆*dgeo*_1985R mutant strains (w1 and w2). (**A**) There are two IS element integration sites of IS*Dge5* and IS*Dge2* on *dgeo*_0524 and *dgeo*_0523, respectively, which encode phytoene synthase. (**B**) PCR detection of nine IS*Dge2* copies in the genome was amplified using the target gene primer sets from a parent strain and ∆*dgeo*_1985R_w1: *dgeo*_1673 (0.98 kb), *dgeo*_2377 (0.76 kb), *dgeo*_2446 (0.75 kb), *dgeo*_2436 (0.94 kb), *dgeo*_2587 (0.75 kb), *dgeo*_2700 (1.05 kb), *dgeo*_2795 (0.88 kb), *dgeo*_2987 (0.98 kb), and *dgeo*_3100 (0.85 kb). Lanes: M, size marker; odd numbers, parent strain; even numbers, ∆*dgeo*_1985R_w1 mutant.

For two non-pigmented colonies from the ∆*dgeo_*1985R strain, an IS*1* family IS*Dge2* element was integrated at the 117th nucleotide of the *dgeo*_0523 gene encoding a phytoene synthase and an IS*701* family IS*Dge5* element was integrated at the 612th nucleotide of *dgeo*_0524 (Figure 4A). IS*Dge2* has the DR sequence ‘CGCGTTTC’ and the TIR sequence ‘GGTAGTGGCTGC’. IS*Dge5* has the DR sequence ‘TCTTC’ and the TIR sequence ‘CTCAGGAGTTGCACCT’. Because nine copies of the IS*Dge2* IS element were located in the *D. geothermalis* genome, PCR was performed to determine the mechanisms of transposition. These nine copies of IS*Dge2* type transposases were still located on their own loci in the genome (Figure 4B). Therefore, we concluded that IS*Dge2* was transposed through a replicative mechanism. To the best of our knowledge, our study is the first to detect the active transposition of an IS*Dge2* of an IS*1* family member in *D. geothermalis*. IS*Dge5* and IS*Dge7* were actively transposed in the cystine importer-disrupted mutant and IS*Dge2* and IS*Dge5* were also transposed in the cystine importer-overexpressed strain. Therefore, as predicted, the unique IS elements were actively transposed due to oxidative stress, resulting in intracellular redox imbalance.

### 3.4. Streptomycin-Resistant Phenotypic Mutation

**Figure 5:**
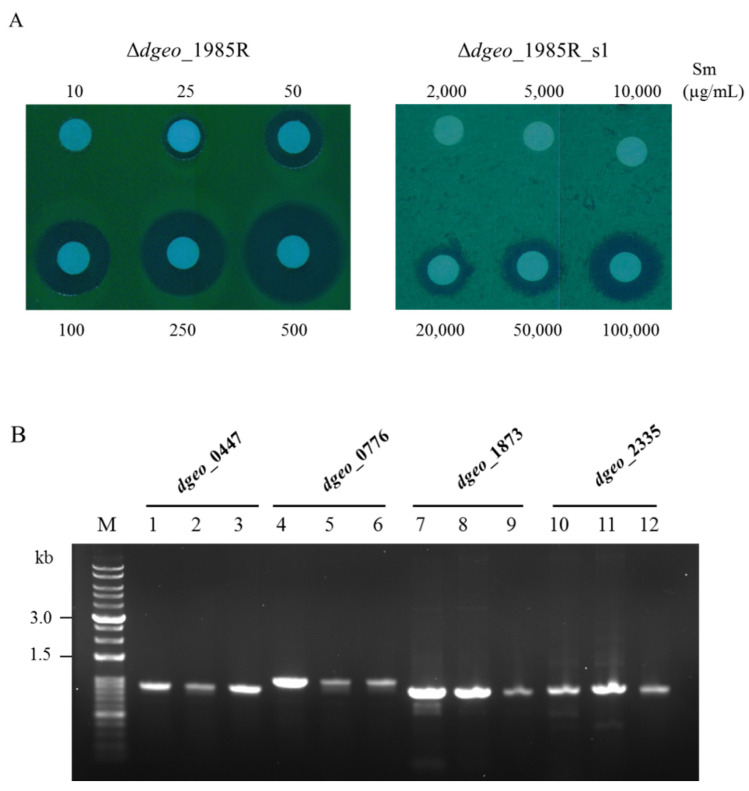
Identification of a streptomycin-resistant mutant from ∆*dgeo*_1985R. (**A**) Measurement of streptomycin MIC values between a parent strain and a SmR mutant (s1). (**B**) PCR detection of four SmR related genes (*dgeo_*0447 (0.9 kb), *dgeo*_0776 (1.05 kb), *dgeo*_1873 (0.9 kb), and *dgeo*_2335 (0.92 kb)) in a genome created using a parent strain and a SmR mutant. Lanes: M, size marker; 1,4,7, and 10, wild-type; 2,5,8, and 11, ∆*dgeo*_1985R parent strain; 3,6,9, and 12, ∆*dgeo*_1985R_s1 mutant.

Additional mutations were also identified using a streptomycin resistance biomarker [28]. This antibiotics resistance-based IS transposition screening could become an effective selection tool to evaluate the oxidative stress response of *D. geothermalis*. *D. geothermalis* has a streptomycin minimum inhibitory concentration (MIC) value of 10 µg/mL and its genome contains four genes that influence streptomycin resistance: two *mthA* (*dgeo*_0447 and 0776) encoding a methylthioadenosine nucleosidase in the S-adenosylmethionine recycling pathway; *rpsL* (*dgeo*_1873) encoding a ribosomal S12 protein, and *rsmG* (*dgeo*_2335) encoding an AdoMet-dependent 16S rRNA methyltransferase. These genes that influence destreptomycin resistance confer different levels of streptomycin resistance upon mutation, resulting in low, moderate, and high MIC values [34]. Interestingly, the Δ*dgeo*_1985R cystine-overexpressed strain exhibited streptomycin-resistant mutants with a frequency similar to that of non-pigment mutant generation after 100 mM H_2_O_2_ treatment. In the case of *Thermus thermophilus*, a streptomycin-resistant mutation occurred via the integration of an IS element into *rsmG* [35]. However, the streptomycin-resistant Δ*dgeo*_1985R mutant did not exhibit any change in the size of the PCR products of four streptomycin resistance genes (Figure 5A,B). Although the streptomycin MIC value of the parent strain Δ*dgeo*_1985R was less than 25 µg/mL, the streptomycin-resistant mutant MIC value exceeded 10,000 µg/mL. After DNA sequencing analysis of the four PCR products, only the *rpsL* gene exhibited a point mutation from adenine to guanine at the 263rd nucleotide resulting in a K88R amino acid substitution. This amino acid substitution in the ribosomal protein S12 confers a strong streptomycin resistance [34]. In this case, the streptomycin-resistant phenotype is caused by a point mutation in the antibiotic resistance gene *rpsL*. Therefore, the streptomycin-resistant mutation of the Δ*dgeo*_1985R mutant via oxidative stress upon H_2_O_2_ treatment was not caused by the transposition of IS elements. Thus, additional studies are required to determine whether transposition is affected by the mutational type of the parent strain.

### 3.5. Gene Expression Levels of Redox Control-Related Genes and Transposases

**Figure 6:**
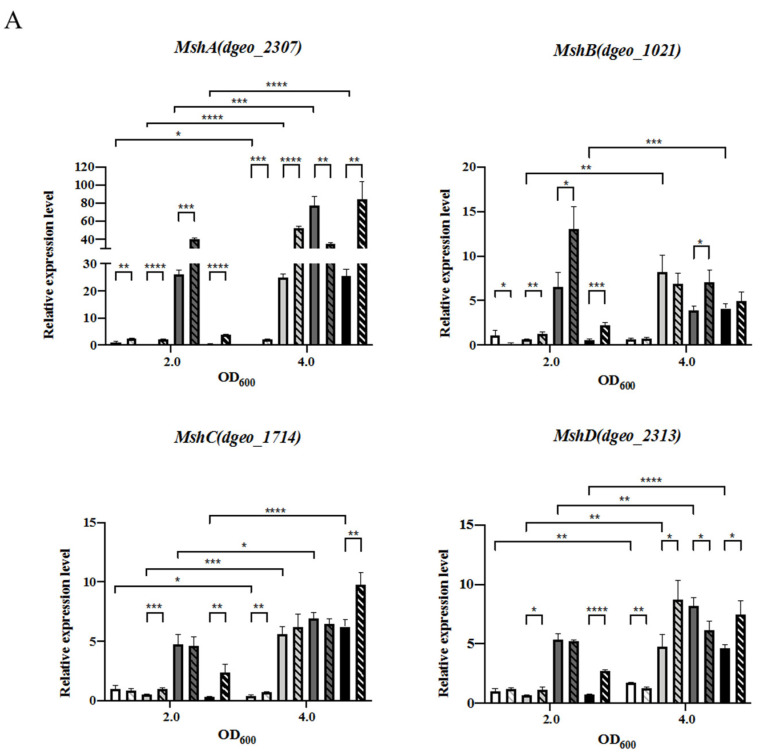

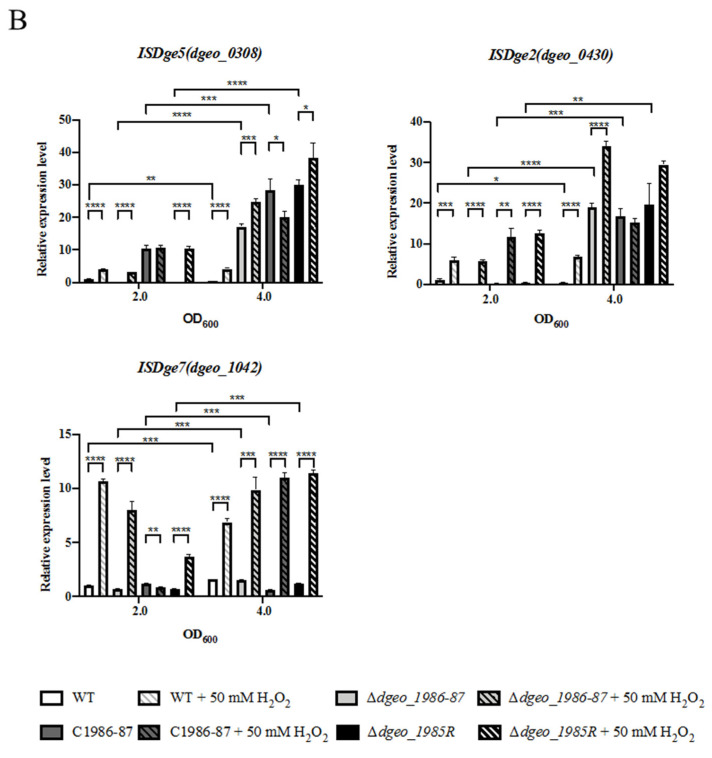
qRT-PCR analysis of four mycothiol biosynthesis genes (**A**) and three IS elements during active transposition (**B**) at two different growth phases in the presence and absence of hydrogen peroxide (50 mM). Pairwise comparisons were conducted using Student’s *t*-test to identify differences between the samples using the Prism^TM^ software. *p* < 0.05 (*), *p* < 0.01 (**), *p* < 0.001 (***), and *p* < 0.0001 (****).

Our transcriptomic analyses indicated that several GCN5-N-acetyltransferase genes were up-regulated in the Δ*dgeo*_1986-87 mutant strain. One of them, *dgeo*_2313, is a putative mycothiol synthase MshD with an 11.2-fold expression (Table 1). Mycothiol (MSH) is a small molecule that acts as a thiol-cofactors for many enzymes and provides protection against oxidative stress by maintaining a reduced intracellular state [36]. The *D. geothermalis* genome contains all MSH biosynthesis pathway genes. Particularly, MshC and MshD were up-regulated in the Δ*dgeo*_1986-87 mutant in the absence of hydrogen peroxide treatment at the late exponential growth phase according to RNA-Seq analysis. Thus, the expression levels of four MSH biosynthesis-related genes were confirmed via qRT-PCR (Figure 6A). At the [E] growth phase, all MSH biosynthesis-related genes were up-regulated on the complementary strain. All four genes were strictly up-regulated by the hydrogen peroxide treatment at the [L] growth phase in the Δ*dgeo*_1986-87 mutant, the complemented strain, and the overexpressed strain. Particularly, MshA (*dgeo*_2307) was up-regulated in response to hydrogen peroxide treatment at the [L] growth phase and its expression levels in all tested mutants exceeded 40-fold except for the wild-type strain. MshB (*dgeo*_1021), MshC (*dgeo*_1714), and MshD (*dgeo*_2313) were up-regulated (5-fold increase) by hydrogen peroxide treatment at the [L] stage. In the case of the cystine importer-complemented strain, the four MSH biosynthesis genes were up-regulated in both the presence and absence of hydrogen peroxide treatment, particularly during the [E] phase. Therefore, MSH may substitute cystine-based thiol in the cystine importer-disrupted mutant and possibly exerts a protective role during oxidative stress, particularly at the [L] stage. Moreover, we sought to quantify the expression of MSH in oxidative stress conditions to assess the functional roles of LMW-thiols as cysteine-derived thiol substitutes in redox-imbalanced conditions.

Three actively transposed IS elements in redox-imbalanced conditions were also detected via qRT-PCR. The transposases (Tpase) of IS*Dge2* and IS*Dge5* were highly up-regulated (over 10-fold increase) at the [L] growth phase both with and without hydrogen peroxide treatment except in the wild-type strain (Figure 6B). This IS*Dge5* induction was consistent with the RNA-Seq data (Table 1). Interestingly, the Tpase of IS*Dge7* was significantly up-regulated (4 to 10-fold increase) in response to hydrogen peroxide treatment at both growth phases. However, in the absence of hydrogen peroxide, this Tpase was not fully induced at both growth phases. The induction levels of Tpase for IS*Dge2* and IS*Dge7* in RNA-Seq data were not substantially detected in the cystine importer-disrupted mutant (Table 1). Nevertheless, these two IS elements, as well as IS*Dge5*, were actively transposed into the target genes (*dgeo*_0523 and *dgeo*_0524) in both the cystine importer-disrupted and -overexpressed strains. Therefore, the active transposition of a particular IS element should be somehow affected by the growth phase and redox-imbalanced conditions.

OxyR is highly induced in a growth phase-dependent manner, mainly via hydrogen peroxide treatment. A single catalase (*katE*) gene was also induced throughout the growth phase and was strongly induced by hydrogen peroxide treatment except in the complemented strains in both growth phases (Figure S4). The OxyR induction of the cystine importer-disrupted mutant and the complemented strain at the [E] stage did not correlate with catalase expression. Therefore, the general oxidative stress response regulator OxyR does not activate catalase induction in *D. geothermalis*. We also tested the expression levels of an oxidative stress response candidate regulator PerR (*dgeo*_2141). Interestingly, this candidate was not sensitive to oxidative stress by hydrogen peroxide treatment at neither the [E] nor the [L] growth phases.

Next, we tested the expression levels of two bacillithiol (BSH) biosynthesis genes, BshA (*dgeo*_1099) and BstA (*dgeo*_1829), as a positive control for antioxidant response in some Gram-positive bacteria such as *Bacillus* sp., *Staphylococcus aureus*, and *D. radiodurans* [2,37]. BshA was highly up-regulated by hydrogen peroxide treatment at both growth phases. Interestingly, BstA was significantly more up-regulated (>2-fold increase) at the [L] stage than at the [E] stage in the absence of H_2_O_2_. Further, in the presence of H_2_O_2_, both the cystine importer-disrupted mutant and the overexpressed mutant exhibited significant gene expression changes (Figure S5). Additionally, based on our transcriptomic analyses, the expression levels of three redox switch-related regulators (HxlR, MarR, and AraC) and a multidrug resistance protein (SugE) were tested (Table 1). Both the HxlR and MarR genes were specifically up-regulated by H_2_O_2_ treatment at the [E] stage. Moreover, both SugE and AraC were significantly up-regulated at both growth phases by H_2_O_2_ treatment (Figure S6).

Our study verified the expression levels of oxidative stress response genes (*oxyR*, *katE*, and *perR*), LMW thiols biosynthesis genes (MSH and BSH), several putative regulators (HxlR, MarR, and AraC), a multidrug resistance protein (SugE), and active transposed IS elements in response to H_2_O_2_ in wild-type and redox-imbalanced mutants. Many target genes were markedly sensitive to oxidative stress at different growth phases and imbalanced redox conditions. However, we did not identify any potential trigger factors for the active transposition of IS elements. Therefore, additional studies are required to identify the redox-sensing regulators that control the related genes to protect intracellular damage and redox imbalances under oxidative stress conditions, which could be responsible for the specific induction of IS element transposition in radiation-resistant bacteria.

## 4. Discussion

Some scholars have recently proposed the replacement of the term “oxidative stress” for “redox biology” because it is an essential process for living organisms, which evolved from signalling processes in many physiological contexts [38]. Both free-living bacteria and intracellular pathogens are exposed to different RONS and RES. The molecular pathways that maintain redox homeostasis in these bacteria constitute a fundamental mechanism that protects them against oxidative stress using enzymatic protection (e.g., catalase, peroxidase, and superoxide dismutase (SOD)) and redox compounds such as redoxins and LMW thiols. GSH has been identified in eukaryotes and Gram-negative bacteria, and many LMW thiols such as MSH, BSH, and ergothioneine (EGT) are found in bacteria. In contrast, archaea exhibit redoxins, EGT, and GSH but not MSH or BSH [8]. Cysteine and coenzyme A are also found in all living organisms. *Actinomycetes* and Gram-positive bacteria have MSH, which actively reacts with RONS and oxidised proteins [36]. Here, a cystine importer-disrupted mutant of *D. geothermalis* exhibited an up-regulation of MSH biosynthesis genes, thus substituting cysteine-derived thiols (either fully or partially) to grant protection against H_2_O_2_ (Figure 6A). Further, BSH biosynthesis also participated in the redox imbalanced responses (Figure S5).

Many regulators that preserve redox-balance act as redox switches. For example, OxyR acts as a thiol-based redox sensor for peroxides and nitrogen oxide in *E. coli* and *Actinomycetes*. PerR is a metal-based peroxide sensor in Gram-positive *Firmicutes*. MarR/OhrR-family regulators act as sensors for organic hydroperoxides and MarR/DUF24-family regulators are sensors for ROS and RES by thiol-oxidation or *S*-alkylation. Spx disulfide-stress redox sensors are also expressed in *Firmicutes*. There are also several extracytoplasmic sigma factors, in addition to their cognate redox-sensitive zinc-associated anti-sigma factors [10,11]. Many of these redox sensors employ conserved cysteine residues for redox-sensing of RONS, HOCl, or RES, and chemical modifications of cysteine residues affected the activation of regulators. Thiol-oxidation also plays an essential role in pathogens, as they have to cope with ROS to defend themselves against the host immune system. Particularly, a DnaK suppressor protein (DksA) regulates the stringent response, central metabolic pathways, and NAD(P)H/NAD(P)^+^ redox balance in *Salmonella* [39]. There are many complex pathways for anti-oxidation defence and gene regulation, and redox-based regulators are classified into three major groups: one-component systems, two-component systems, and a heterogeneous group of flavin-based photosensors [11]. The general oxidative stress response regulator OxyR is a known global activator in bacteria [9,40]. Nevertheless, when OxyR was highly expressed by H_2_O_2_ treatment, H_2_O_2_ scavenging enzymes (catalase and SOD) and a DNA protecting protein (Dps) were not proportionally induced in *D. geothermalis* (Figure S4) [21]. Recent transcriptomic and proteomic analyses have provided important insights into the expression and regulation of genes in *Deinococcus* under different stress conditions [41,42,43,44]. The gene expression levels of many gene categories were affected in the Δ*dgeo*_1986-87 strain (Table 1). Interestingly, the mode of action of some transcriptional regulators might be mediated by thiol oxidation sensors. For example, the AraC family *dgeo*_2329, MarR type regulator *dgeo*_1148, HxlR family dgeo_0527, and an uncharacterised multi-resistant SugE family *dgeo*_1956 were up-regulated (8.38-, 3.18-, 3.35-, and 6.36-fold increases, respectively) in a cystine importer-disrupted mutant (Figure S6). However, the relationship between redox-sensing and gene expression regulators is unclear and therefore additional studies are required to characterise their functional roles in *D. geothermalis*.

In *Lactobacillus sanfranciscensis*, a cystine importer-disrupted mutant showed high up-regulation of the *opp* operon for methionine transporter, as well as two redox-sensing proteins, *spxA* and *nrdH* [45]. However, NrdH redoxins were highly up-regulated by H_2_O_2_ treatment at the [E] phase in the wild-type strain, but its expression levels were not affected in either growth phase or in the presence of hydrogen peroxide in the *D. geothermalis* cystine importer-disrupted mutant (Figure S7). In the case of *Saccharomyces cerevisiae* (i.e., a eukaryotic model), disruption of a cystine importer resulted in up-regulation of several genes encoding proteins associated with sulphur regulation, cellular respiration, and general transporters [46]. Disruption of a cystine importer in the Δ*dgeo*_1986-87 strain of *D. geothermalis* resulted in up-regulation of GCN5 family proteins, some ABC transporters, sigma factors, enzymes, and regulators, as well as an IS*Dge5* transposase (Table 1). However, peptide ABC transporters such as the *opp* operon (*dgeo*_2122), two regulators (*dgeo*_2619, a RpiR family regulator, and *dgeo*_2515, and a putative regulator), and several enzymes were down-regulated (Table 2). Therefore, although the expression of redox imbalance-related genes did not correlate among different species, there are many possibilities for network regulation between the cystine importer-based redox imbalance and their responding genes.

The bacterial genome contains an abundance of transposable elements (TE) including ISs [23]. Recent bacterial mobilome approaches have been applied for the detection of these TE elements [24,25]. IS transposition is a transposase-mediated process that only occurs in a minority of cells within a bacterial culture [47]. Therefore, there are likely many pathways for active transposition under redox-based regulation. Nevertheless, few case studies of IS transposition induction have been conducted so far. Mitomycin C and gamma-irradiation resulted in IS transposition in *D. radiodurans* [48,49,50,51]. When the translation error rate was enhanced by mutation of *ygjD* and *rpsL* encoding the universal tRNA and rRNA modifying proteins, respectively, or under high glucose growth conditions, active transposition of an IS*1* family member was found in *Escherichia coli* [47,52]. Additionally, nutritional stress and host factors have been implicated in the transposition of ISs and TEs [53,54]. However, the relationship between the intracellular redox imbalance and the active transposition of ISs is still not fully understood.

A special DNA-binding protein disruption in our previous work resulted in active transposition of a certain family member IS element under oxidative stress conditions. Disruption of putative Dps protein *dgeo*_0257 resulted in the movement of both IS*Dge5* and IS*Dge7* elements to other sites. Particularly, IS*Dge7* was integrated into a gene encoding phytoene desaturase under the carotenoid biosynthesis pathway, which led to the discovery and selection of a non-pigmented colony [29]. In another case, disruption of the putative LysR family regulator *dgeo*_2840 resulted in the integration of the IS*Dge6* element into the same gene encoding phytoene desaturase [30]. Both of these DNA-binding proteins likely participated in the transposition of the particular IS element. We first proposed that IS transposition was triggered by the redox imbalances of the cystine importer-disrupted mutant and its complement strain. The Δ*dgeo*_1986-87 and Δ*dgeo*_1985R mutant strains showed the transposition of IS*Dge5*/IS*Dge7* and IS*Dge5*/IS*Dge2*, respectively. Interestingly, the transposition of IS*Dge5*/IS*Dge7* involves the same IS elements in a putative Dps gene *dgeo*_0257-disrupted mutant. The Dps1 gene *dgeo*_0281-disrupted mutant has an IS transposition of IS*Dge5* on the same target gene (unpublished data). Therefore, Dps (i.e., proteins that protect DNA from oxidative damage) are also somehow involved in the transposition of unique IS elements. There might also be a correlation between redox imbalance and Dps deficiencies in starving cell with identical IS element transposition. In turn, this may be mediated by a certain thiol-sensing and oxidation state-responding regulator or direct sensing of oxidation state on transposase activation. Although Dps bind to DNA via a non-specific action mode similar to host factors such as HU, H-NS, IHF, and others, Dps deficiency may induce the transposition of a specific IS element via unique transposase activation. Interestingly, according to the RNA-Seq analysis of the Δ*dgeo*_1986-87 mutant, the IS*Dge5* element was highly up-regulated (>4.5-fold increase) and active transposition was directly detected under oxidative stress conditions (Table 1 and Figure 3). However, IS*Dge2* was only actively transposed in the Δ*dgeo*_1985R mutant. There are many open questions regarding the activation of IS element transposition in response to specific DNA-binding protein disruption and redox imbalance caused by the cystine importer under oxidative stress conditions.

In addition to the transposition of IS*4*, IS*5*, IS*630*, and IS*200*/IS*605* into *thyA* through gamma-irradiation in *D. radiodurans* [49], another study recently reported that heat stress enhanced the transposition of multiple ISs via *sigX*-dependent stress response in *Geobacillus kaustophilus* [55]. In this case, the IS*4*, IS*701*, IS*1634,* and IS*Lre2* family members were transposed. Multiple transposition of the IS*1*, IS*4*, IS*5*, and IS*701* families have been reported in carotenoid biosynthesis genes of *D. geothermalis* in various physiological states (Figure 3, Figure 4 and Figure 6B) [29,30]. Therefore, whether the IS transposition is a random occurrence or a specific occurrence depends on DNA-binding proteins and redox stress-responding proteins involved in redox control. The DdrO/IrrE system is only present in *Deinococcus* bacteria and is a well-known gene expression control system that becomes activated during the gamma-irradiation and desiccation response (RDR) [56]. If the redox signalling pathway induces the regulation of particular regulatory proteins such as IrrE-DdrO in response to the zinc released from zinc-chelated proteins upon ROS attack, zinc ions activate protease IrrE and the activated IrrE cleaves the transcriptional repressor DdrO, thus inducing the expression of target genes involved in RDR regulons [57]. Assuming that the induction of IS element transposition is similarly regulated, it would be possible to determine the transposition of particular IS elements that act selectively in redox imbalance conditions. Transposase activity and stability are exciting topics that remain poorly understood, and therefore future studies should explore the relationship between IS element transposition trigger factors and redox-sensing regulators.

## Figures and Tables

**Table 1:** List of up-regulated genes (>3.0-fold increase) in the cystine importer-disrupted mutant.

Category	Gene	Fold Change	Function
Transposase (IS*Dge5*, IS*701* family)	*dgeo*_0308	4.82	Transposase
	*dgeo*_0464	3.71	Transposase
	*dgeo*_0925	3.74	Transposase
	*dgeo*_1807	6.15	Transposase
	*dgeo*_2205	3.92	Transposase
	*dgeo*_2430	4.93	Transposase
	*dgeo*_2823	4.55	Transposase
	*dgeo*_2659	4.65	Transposase
Sigma factor	*dgeo*_1346	4.13	Sigma 70
	*dgeo*_1519	3.26	Sigma factor
Regulator	*dgeo*_0527	3.35	HxlR family
	*dgeo*_1148	3.18	MarR family
	*dgeo*_1956	6.36	SugE family
	*dgeo*_2329	8.36	AraC family
ABC transporter	*dgeo*_0543	3.34	ABC transporter
	*dgeo*_0647-48	6.96–7.06	ABC transporter
	*dgeo*_0954-56	4.20–9.76	Chromate transporter
	*dgeo*_1413	5.91	ABC transporter
	*dgeo*_1805	8.03	ABC transporter
	*dgeo*_2443	4.69	Iron permease
	*dgeo*_2581-82	3.60–3.25	cation transporter
MFS transporter	*dgeo*_0249	3.75	MFS transporter
	*dgeo*_0530	6.41	MFS efflux
	*dgeo*_1968	5.57	MFS transporter
	*dgeo*_2330	3.22	MFS efflux pump
GCN5 family	*dgeo*_0369-70	9.60–12.53	GCN5 family regulator
	*dgeo*_2125	3.73	GCN5 family acetyltransferase
	*dgeo*_2313	11.2	GCN5 family acetyltransferase
Enzyme	*dgeo*_0071	5.01	Peptidase M29
	*dgeo*_0334	4.68	Carboxypeptidase
	*dgeo*_0431	5	Phage integrase
	*dgeo*_0528	8.8	NADH dehydrogenase
	*dgeo*_0570	3.68	Proline tRNA ligase
	*dgeo*_0824	3.34	Exonuclease
	*dgeo*_0909	3.45	NADH-quinone oxidoreductase
	*dgeo*_1337	6.43	Methylenetetrahydrofolate reductase
	*dgeo*_1407-08	7.49–9.32	Ferredoxin nitrite reductase/sulfate adenylyltransferase
	*dgeo*_1714	10.78	Cysteine tRNA ligase
	*dgeo*_2583	3.49	NrdH redoxin
	*dgeo*_2681	3.63	Zn dependent hydrolase
	*dgeo*_2801	3.14	WYL domain protein

**Table 2:** List of down-regulated genes (<0.3-fold decrease) in the cystine importer-disrupted mutant.

Loci	Gene	Fold	Function
Chromosome	*dgeo*_1056	0.22	Nuclease inhibitor
	*dgeo*_1081	0.27	3-hydroxyisobutyrate dehydrogenase
	*dgeo*_1535-36	0.17	Thioesterase/pterin dehydratase
	*dgeo*_2122	0.07	Peptide ABC transporter
	*dgeo*_2245	0.27	Citrate synthase
Plasmid 1	*dgeo*_2704	0.25	Cytochrome D subunit
	*dgeo*_2619	0.2	RpiR family regulator
	*dgeo*_2561	0.25	Peptidase C39
	*dgeo*_2515	0.29	DNA binding protein
Plasmid 2	*dgeo*_3003-04	0.12	Thiamine biosynthesis/ferric reductase

## Data Availability

Data are contained within the article or Supplementary Material.

## Supplementary Materials

The following are available online at www.mdpi.com/article/10.3390/antiox10101623/s1. Figure S1: Growth curves of WT, ∆dgeo_1986-87, complement ∆dgeo_1986-87, and ∆dgeo_1985R. Figure S2: Comparison of viability between the [E] phase (OD600 2.0) and the [L] phase (OD600 4.0). Figure S3: PCR detection of ten ISDge5 copies using the target gene primer sets from ∆dgeo_1986-87_w1. Figure S4: qRT-PCR analysis of OxyR, catalase (KatE), and a putative redox-sensing regulator PerR. Figure S5: qRT-PCR analysis of two selected BSH synthesis genes, BshA (dgeo_1099) and BstA (dgeo_1829). Figure S6: qRT-PCR analysis of four selected regulating genes, HxlR (dgeo_0527), MarR (dgeo_1148), SugE (dgeo_1956), and AraC (dgeo_2329). Figure S7. qRT-PCR analysis of two redoxin genes, NrdH redoxin (dgeo_2583) and redoxin (dgeo_2729) as a neighbour gene of catalase (dgeo_2728, KatE).

## Abbreviations

BSH	bacillithiol
Dps	DNA-binding protein for DNA protection in starved cell
IS	insertion sequence
MSH	mycothiol
RES	reactive electrophilic species
RONS	reactive oxygen/nitrogen species
