# Supplementary material for: Influence of Redox Imbalances on the Transposition of Insertion Sequences in Deinococcus geothermalis
Source: Antioxidants (Basel). 2021 Oct 15;10(10):1623. doi: 10.3390/antiox10101623 (PMC8533066; doi:10.3390/antiox10101623)
Supplement: Supplementary file 1 [file antioxidants-10-01623-s001.zip › antioxidants-1355951-Supplementary.pdf]

**Supplementary data (Ye et al., 2021)**

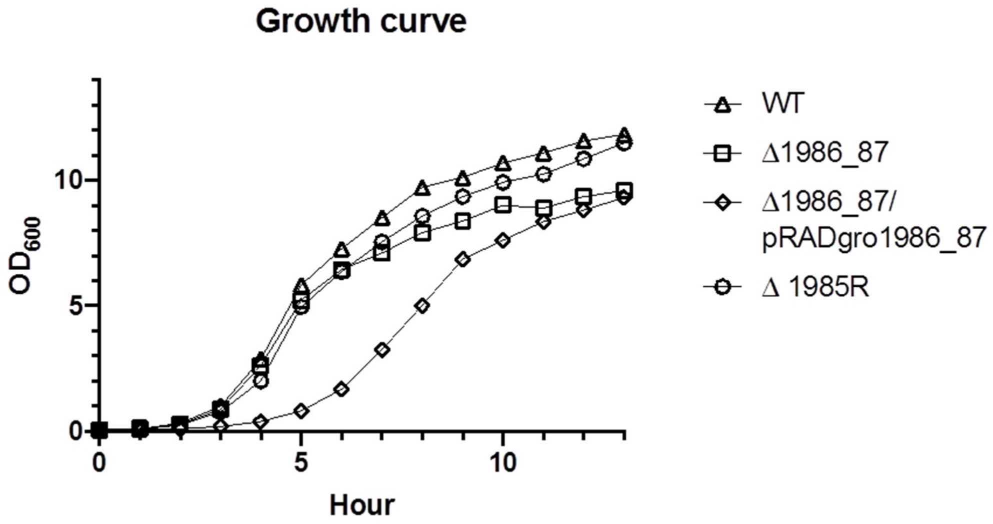


**Figure S1.** Growth curves of WT, Δ*dgeo*_1986-87, complement Δ*dgeo*_1986-87, and Δ*dgeo*_1985R. Growth curve could be divided into two groups, which are WT/ Δ*dgeo*_1985R and Δ *dgeo*_1986-87. Δ*dgeo*_1986-87 was delayed and lower maximal absorbance since middle exponential growth phase. The complement strain was delayed by chloramphenicol growth inhibition and maximal growth was approached till lower maximal absorbance.

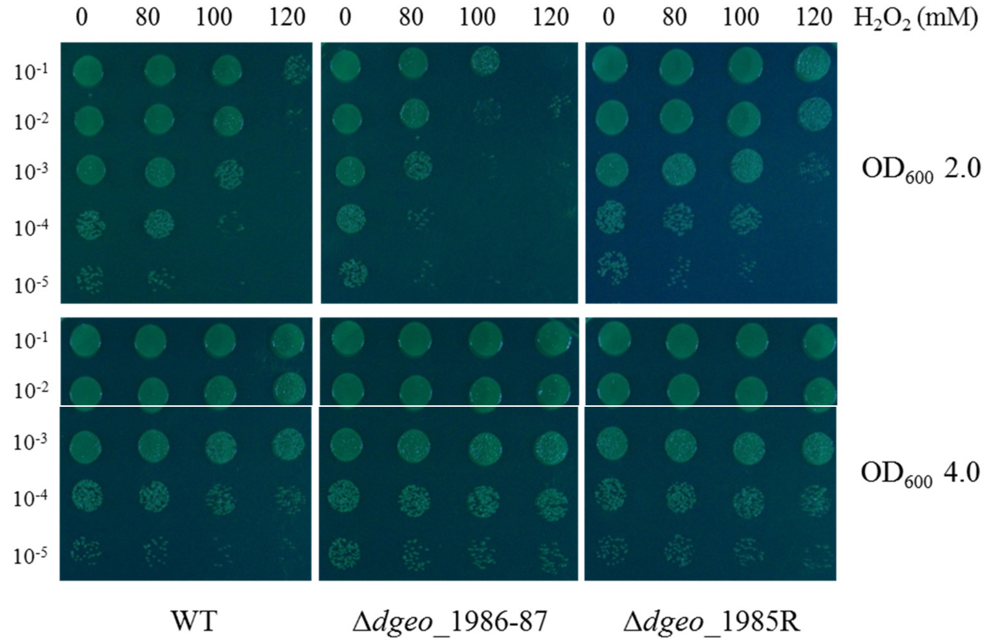


**Figure S2.** Comparison of viability between [E] phase ($OD_{600}$ 2.0) and [L] phase ($OD_{600}$ 4.0) among wild-type, Δ*dgeo*_1986-87, and Δ*dgeo*_1985R by the different final concentration of hydrogen peroxide.

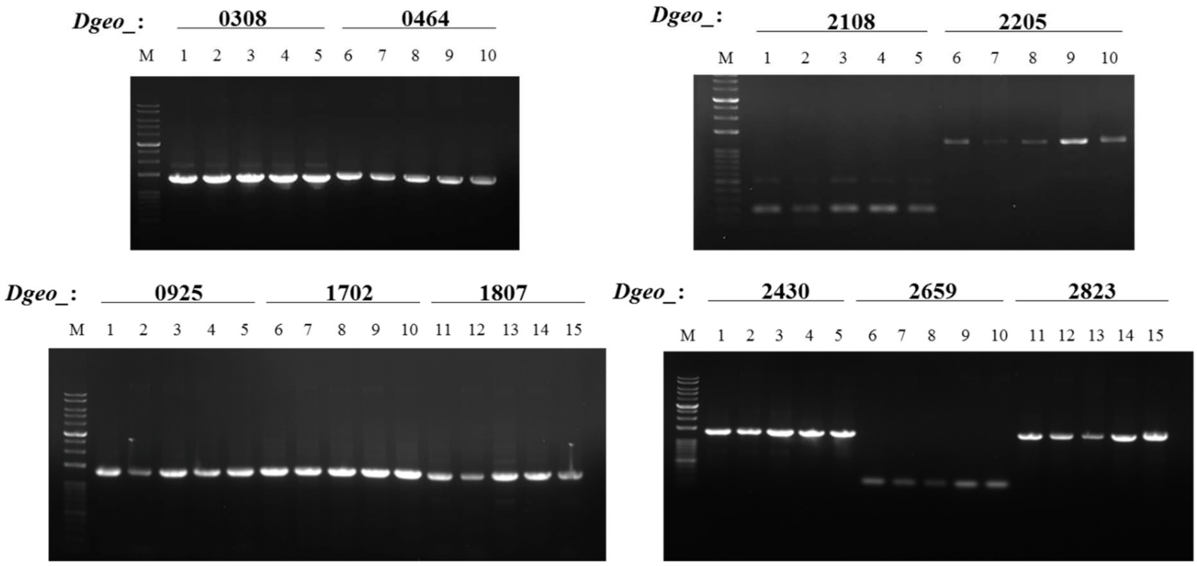


**Figure S3.** PCR detection of ten IS*Dge5* copies was amplified by the target gene primer sets from Δ*dgeo*_1986-87_w1.

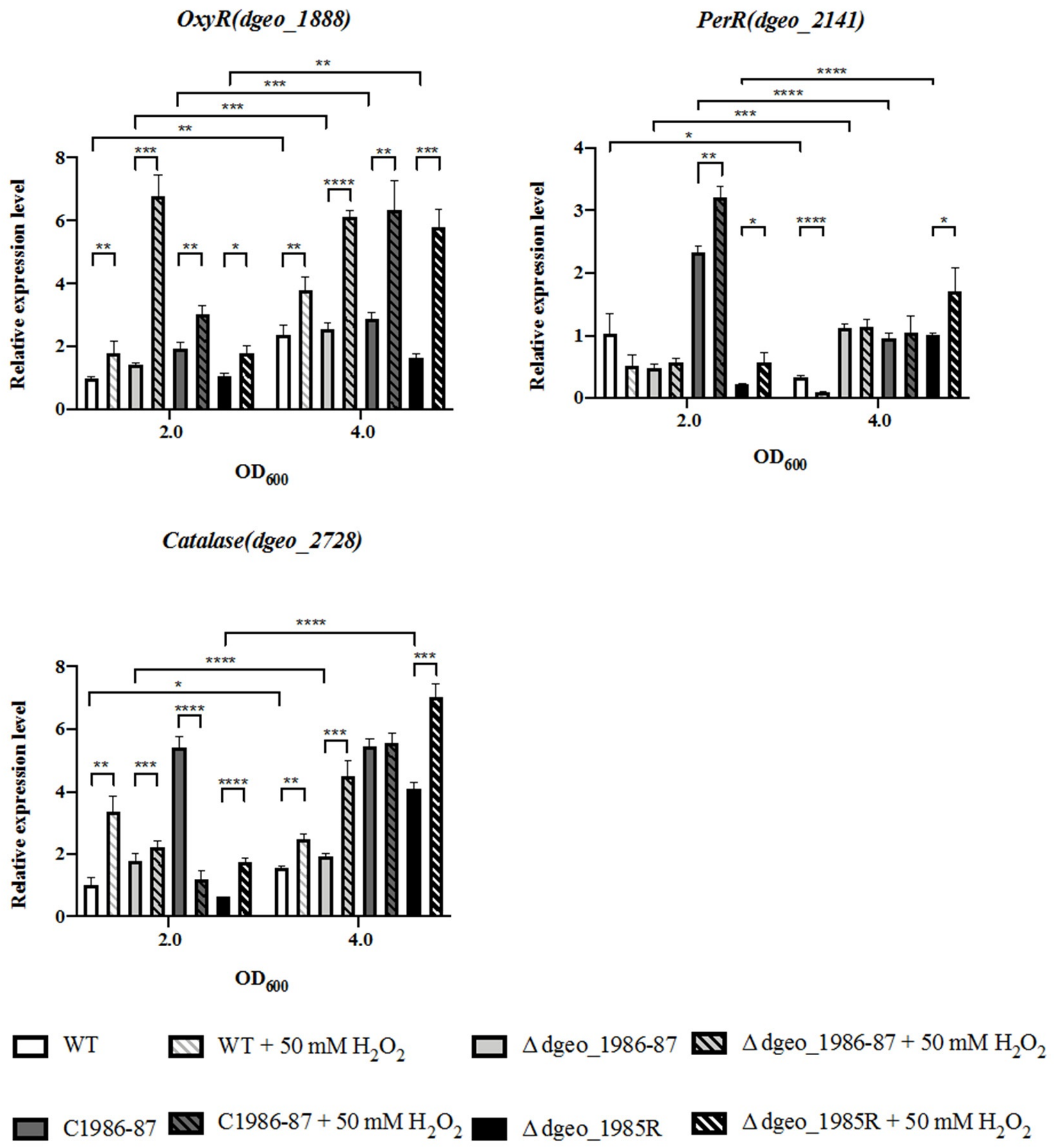


**Figure S4.** qRT-PCR assay for expression levels of OxyR, catalase (KatE), and a putative redox-sensing regulator PerR at two different growth phases with present or absent hydrogen peroxide of 50 mM in this study. The statistical analysis was done by probability *t*-test for differences between the samples in Prism™ program (ver. 8.0) and it was considered significant at $p < 0.05$ (*), $p < 0.01$ (**), $p < 0.001$ (***), and $p < 0.0001$ (****).

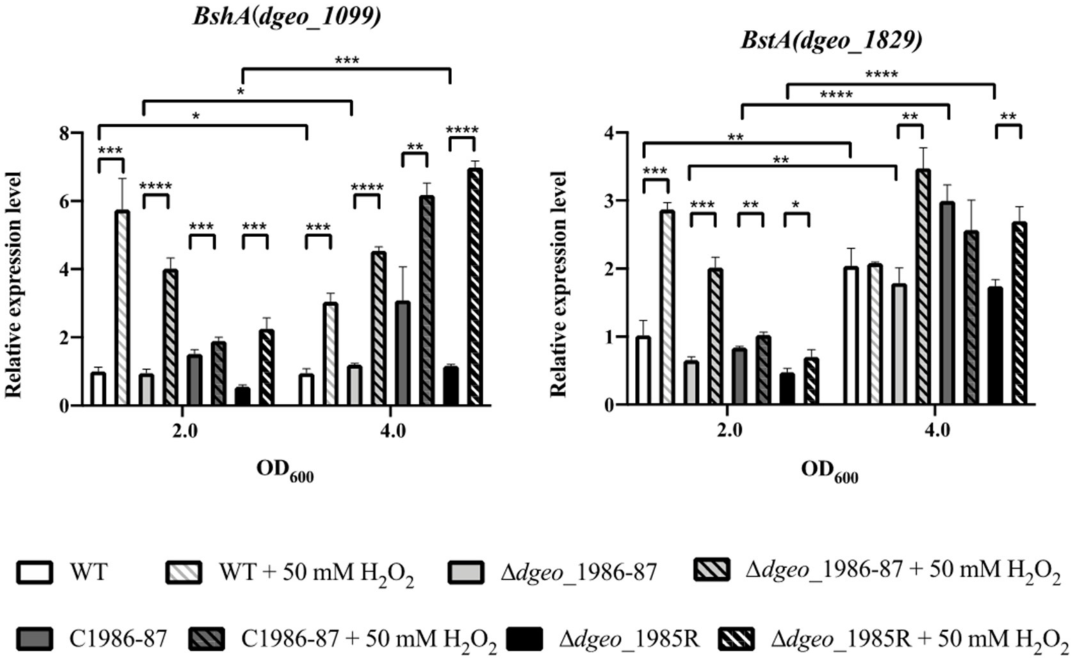


**Figure S5.** qRT-PCR assay for expression levels of selected two BSH synthesis involved genes, BshA (*dgeo*_1099) and BstA (*dgeo*_1829) at two different growth phases with present or absent hydrogen peroxide of 50 mM in this study.

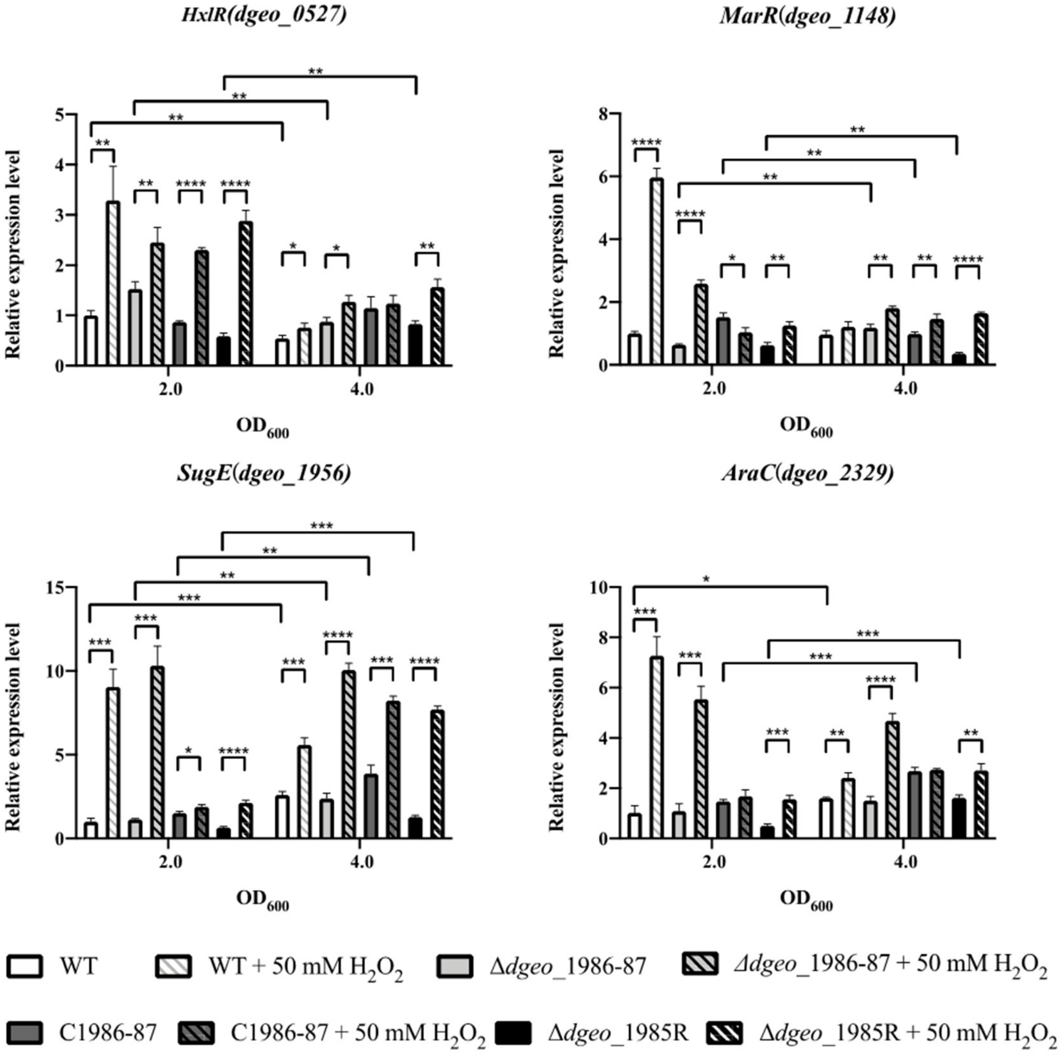


**Figure S6.** qRT-PCR assay for expression levels of selected four regulating genes at two different growth phases with present or absent hydrogen peroxide of 50 mM in this study. Based on the transcriptomic study, expression levels of HxlR (*dgeo*_0527), MarR (*dgeo*_1148), SugE (*dgeo*_1956), and AraC (*dgeo*_2329) were tested.

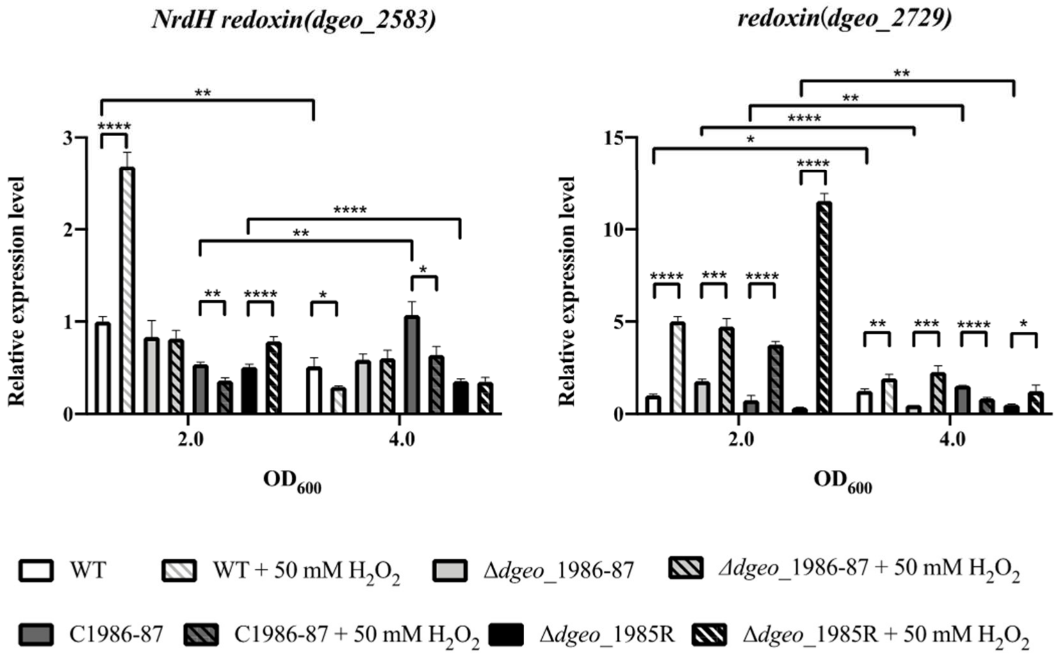


**Figure S7.** qRT-PCR assay for expression levels of two redoxin genes such as NrdH redoxin and a catalase neighbor redoxin at two different growth phases with present or absent hydrogen peroxide of 50 mM.
